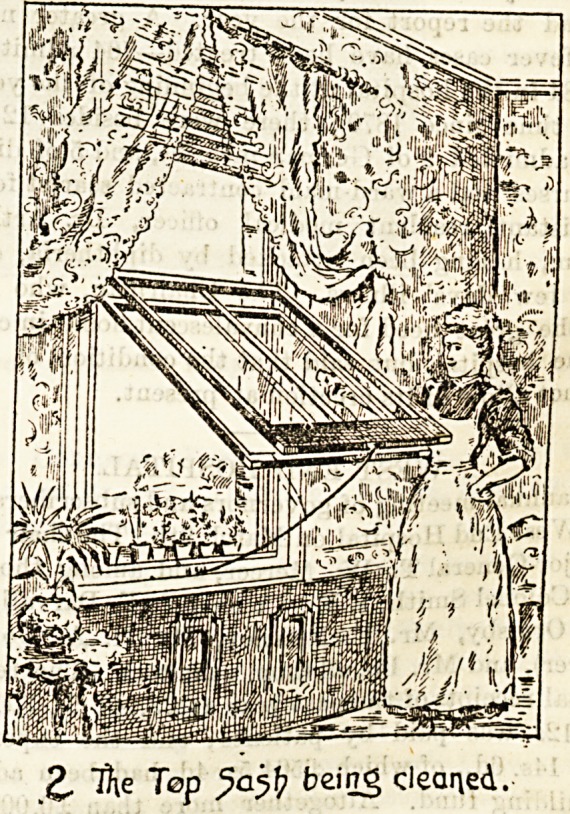# Practical Departments

**Published:** 1894-03-17

**Authors:** 


### PRACTICAL DEPARTMENTS.

### A NEW WINDOW.

The loss of life occurring every year in consequence of our present system of window-cleaning has really assumed serious proportions, in spite of the provisions of the law for its prevention, and the subject has been before discussed in The Hospital.

More than one "safety" window has been brought before the notice of the public of recent years, with a view of preventing so needless a sacrifice of lives by facilitating cleaning operations. The difficulty, however, of carrying out practically any plan of this sort without interfering to any extent with the appearance of the window frame, from an artistic point of view, or with its durability, has been hard to overcome, and the ugliness of the safety frames hitherto invented has been sufficient reason to account for their non-adoption. But these objections cannot be urged against the window shown in the accompanying sketches, the patent of the National Accident Prevention Window Company, Limited. There is nothing the least unsightly in these frames, which in appearance, except when actually in process of cleaning, differ nothing from the ordinary sash window, and slide in the usual way. It is, in fact, a perfect combination of a sliding and inward opening window, and in addition to its primary object—the prevention of loss of life—the saving of time (or labour) in window cleaning, glazing, painting, &c., must be very considerable. When opened inwards, such process involves no detrimental strains upon the joints of the sashes whatever, thus ensuring durability. This desirable result is achieved owing to its unique governing principle, viz.: Top suspension. During the process of opening each sash inwards, it is suspended at the top—the hinging arrangement being independent of the joints.

1. Mode of Cleaning the Bottom Sash.

We understand the inventor is an architect, who has worked at the subject theoretically and practically for ten years. He has effected the inward opening of a sliding sash, casement-fashion; also upon centres like a looking glass; and downwards like the tail board of a cart. He has found neither of these principles to be satisfactory, constructionally speaking, for the following reasons:—

The casement system is wrong theoretically, because of the unavoidable tendency of the sash to drop out of square; and consequently to give trouble when it is desired to slide it up or down. The centre-hung principle prevents window gardening and the fixing of outside sun blinds, and is awkward in working; besides which it causes the sash stiles after a little time to become more or less "bowed" or bent. The downward opening arrangement causes great strain upon the meeting-rail joints.

In a medical contemporary, we notice in a comment upon the subject of deaths from window cleaning, that the wholesale abolition of the sash window is suggested as the only remedy for the evil. The window alternatively proposed is no doubt excellent, but casement windows have undoubted inconveniences, and the difficulty it seems to us may be easily solved by the adoption of the modified form of sash window invented by the National Accident Prevention Company.

The first illustration shows the lower sash of the "N.A.P." window in position for cleaning, which, it will be seen, is quite easily accomplished. The stay-bar supporting the sash is in no way a fixture, and one is sufficient for any number of windows, being carried from one to another by the cleaner. The second illustration shows the top sash attached to the stay bar just mentioned.

The method of placing the sashes in position for cleaning is extremely simple. One of two distinct plans may be followed in fitting these frames: the "Top Bolt Patent," intended chiefly for the alteration of existing windows, or the "Divided Sash Stile Patent," applied more particularly to new windows. Both are worked on the principle of top suspension. In the first plan the beads of the window frame are hinged for about one-half their height. Bolts are then affixed to the ends of the top rails of both sashes, so that when the inside beads are open, and the bolts shot, the bottom sash can be pulled inwards from the bottom to be cleaned, and secured to the supporting stay-bar, which is fixed in position in a second, as shown in the drawings. "The top sash, having been previously cleaned on the inside, can be lowered and pulled inwards in the same manner for outside cleaning."

If the "divided sash" plan is followed, instead of the beads being hinged, the sides of the sashes themselves are divided vertically, and have a gun-metal hinge at the top joining the two pieces thus divided, and a simple device for securing them is used at the bottom. When the securing device is undone, the sashes can be pulled inwards as already described, and in either case be replaced with perfect ease.

We are not surprised to learn that these windows are becoming extensively adopted by well-known architects, and are already being used in many new buildings. Both at the Imperial Institute and by the War Office they have been used, and Mr. Alfred Waterhouse, R.A., amongst others, is ordering their use in buildings now in progress. We understand that the authorities of one of our largest London Hostals are arranging for existing window frames to be altered in accordance with the "N.A.P." principle, and for institution use they will assuredly prove very valuable, as the saving of labour will be considerable, in addition to that of lives and limbs, where window cleaning is required on an extensive scale.

2. The Top Sash being cleaned.

Our illustrations are given by kind permission of the proprietors, the National Accident Prevention Window Company, Limited, at whose offices, 34, New Bridge Street, E.C., specimen windows may be seen and full particulars obtained.

## Figures and Tables

**1. f1:**
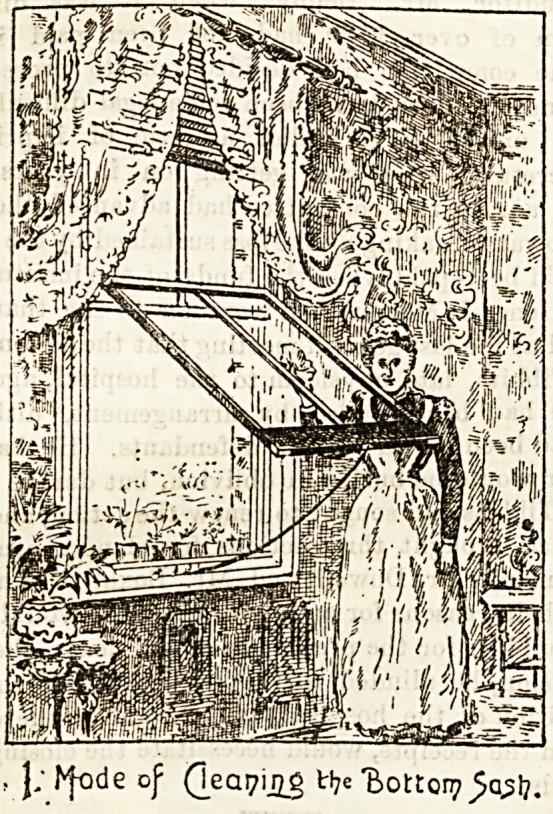


**2 f2:**